# Artificial intelligence‐assisted tracheal intubation in humans: a prospective observational study of diagnostic accuracy

**DOI:** 10.1111/anae.70063

**Published:** 2025-11-17

**Authors:** Alexander Fuchs, Aline Raeber, Ricarda Lippuner, Lea Weber, Yevheniia Borysenko, Markus Huber, Robert Greif, Thomas Riva

**Affiliations:** ^1^ Department of Anaesthesiology and Pain Medicine, Inselspital Bern University Hospital Bern Switzerland; ^2^ Medical Faculty University of Bern Bern Switzerland

**Keywords:** airway management, artificial intelligence, tracheal intubation, videolaryngoscopy

## Abstract

**Introduction:**

larynGuide™ is a novel assistive software integrated with the C‐MAC^®^ videolaryngoscope, which provides guidance during laryngoscopy and advises on tracheal tube position. This first in‐human study evaluated the accuracy and reliability of larynGuide compared with the judgment of the airway operator.

**Methods:**

This prospective, single‐centre, investigator‐initiated, observational study included adult patients undergoing elective surgery requiring general anaesthesia with tracheal intubation. After informed consent and standardised induction of anaesthesia, laryngoscopy and tracheal intubation were performed with a C‐MAC^®^ videolaryngoscope with a Macintosh blade by a board‐certified anaesthetist. larynGuide ran on a second screen, visible only to the study team but blinded to the airway operator. After tracheal intubation attempts, the airway operator confirmed tracheal tube placement visually and with capnography. The primary outcome was the real‐time accuracy of larynGuide in identifying correct tracheal tube placement.

**Results:**

We enrolled 132 patients, of whom 110 were analysed. Of 108 patients with correctly placed tracheal tubes, larynGuide identified 102 (sensitivity 0.94, 95%CI 0.88–0.98). In six patients, the software misclassified tracheal tube position: two false negatives (i.e. the software advised a failed tracheal intubation despite correct placement); and four patients with no feedback. Among two patients with unsuccessful tracheal intubation due to oesophageal tube placement at the first attempt, larynGuide detected one.

**Discussion:**

This first in‐human study has established the feasibility of AI‐guided real‐time tracheal intubation using larynGuide. The software showed promising sensitivity, while specificity was limited. Videolaryngoscopy image quality issues, including fogging and poor visibility, impaired the performance of the software.

## Plain Language Summary

larynGuide™ is a new computer program that works with a special camera tool called the C‐MAC^®^ videolaryngoscope. It helps doctors see the throat more clearly and gives advice on where to place a breathing tube. This study tested how accurate and reliable larynGuide was compared to the doctor's own judgment. The study included adults having planned surgery that needed general anaesthesia and a breathing tube. After patients gave permission, a trained doctor used the C‐MAC^®^ videolaryngoscope to place the breathing tube. The larynGuide software ran on a separate screen that only the research team could see. The doctor did not see the software's advice. After putting in the breathing tube, the doctor checked that it was in the right place using sight a test called capnography that measures carbon dioxide in the patient's breath. The main goal was to see how often larynGuide correctly identified the right tube placement. In total, 132 patients joined the study, and 110 were included in the final results. Of the 108 patients who had their breathing tubes correctly placed, larynGuide also showed correct placement in 102 of them. This means the software was right about 94% of the time. In six cases, the software was wrong or did not give an answer. In two patients where the tube went into the wrong place (the food pipe instead of the windpipe), larynGuide identified one of them. This was the first study to test using larynGuide in real people. The software worked well most of the time and showed that computer help during breathing tube placement is possible. However, it did make some mistakes, especially when the camera view was blocked or blurry.

## Introduction

Tracheal intubation success depends on the airway operator's expertise and experience in laryngoscopy. Less experienced clinicians may have to perform tracheal intubation in emergencies. This could lead to situations where tracheal intubation is not possible, posing potential safety risks to patients. In addition, successful performance is hampered by complex anatomical structures, abnormalities of the upper airway and airway trauma [1].

Failed tracheal intubation or undetected oesophageal intubation can jeopardise patients and cause morbidity and mortality [2]. Inability to ventilate the patient's lungs could result in hypoxaemia, potentially leading to neurologic damage, cardiovascular complications and death [3]. The incidence of unexpected difficult laryngoscopy varies between 5% and 10%, and correlates with an impaired view of the glottis [4, 5]. Videolaryngoscopy has been shown to be superior to direct laryngoscopy by minimising accidental oesophageal intubation [6, 7] in normal airways, rapid sequence intubation and difficult tracheal intubation [8, 9]; improving first‐attempt tracheal intubation success rates [10, 11]; reducing the risks of complications (i.e. hypoxaemia, hypotension or death) [6]; and reducing the incidence of upper airway trauma [8, 9]. While videolaryngoscopy improves glottic visualisation and tracheal intubation success, its effectiveness is influenced by a learning curve that may hinder optimal use by less experienced operators [12].

Newly developed assistive software based on artificial intelligence (AI), larynGuide™ (aiEndoscopic, Zurich, Switzerland), runs on the C‐MAC^®^ (Karl Storz, Tuttlingen, Germany) videolaryngoscope platform. It enables guided videolaryngoscopy by identifying anatomical structures and provides guidance on the correct placement of the tracheal tube, specifically whether it is placed in the trachea or not. Such a clear decision is the key message after the placement of a tracheal tube. Its precursor, the REALITI device (ETH Zurich and UZH, Zurich, Switzerland), showed successful robotic‐assisted tracheal intubation in manikins by less experienced healthcare personnel, indicating that novices were able to intubate tracheas as fast as experienced anaesthesia providers [13], but this could not be replicated in humans.

larynGuide may provide utility in scenarios where less experienced healthcare providers are tasked with performing tracheal intubation; however, its real‐time accuracy in human patients has not yet been established. Therefore, the aim of this first in‐human prospective diagnostic accuracy study was to evaluate the real‐time diagnostic accuracy of the AI‐based larynGuide software in identifying correct tracheal tube placement during videolaryngoscopy compared with the clinical judgment of experienced airway providers. We hypothesised that the sensitivity of larynGuide in identifying correct tracheal intubation would be at least 95%.

## Methods

This was a prospective, single‐centre, investigator‐initiated observational trial conducted at the Inselspital, Bern University Hospital. Following ethical approval and prospective registration, patients scheduled for elective surgery requiring tracheal intubation were screened. Inclusion criteria were age ≥ 18 y and ASA physical status 1–3. We did not study patients meeting any of the following criteria: expected impossible mask ventilation; high risk of aspiration requiring rapid sequence intubation; intracranial surgery; contraindications for tracheal intubation using a C‐MAC videolaryngoscope with a Macintosh blade 3 or 4; limited knowledge of the German language; or declining participation. We obtained written informed consent from all patients before enrolment.

The study was designed intentionally to evaluate the safety and diagnostic accuracy of the larynGuide software in identifying correct tracheal tube placement under clinical conditions before a randomised controlled trial aimed at assessing its full guidance capabilities during tracheal intubation. This study was not intended to assess the software as a replacement for standard methods for confirming tracheal intubation, such as capnography, but rather as a potential adjunct in airway management. larynGuide utilises a custom convolutional neural network trained on an extensive dataset of videolaryngoscope tracheal intubation videos to identify key anatomical landmarks and provide real‐time procedural guidance, without relying on external physiological signals, such as capnography.

**Figure 1:**
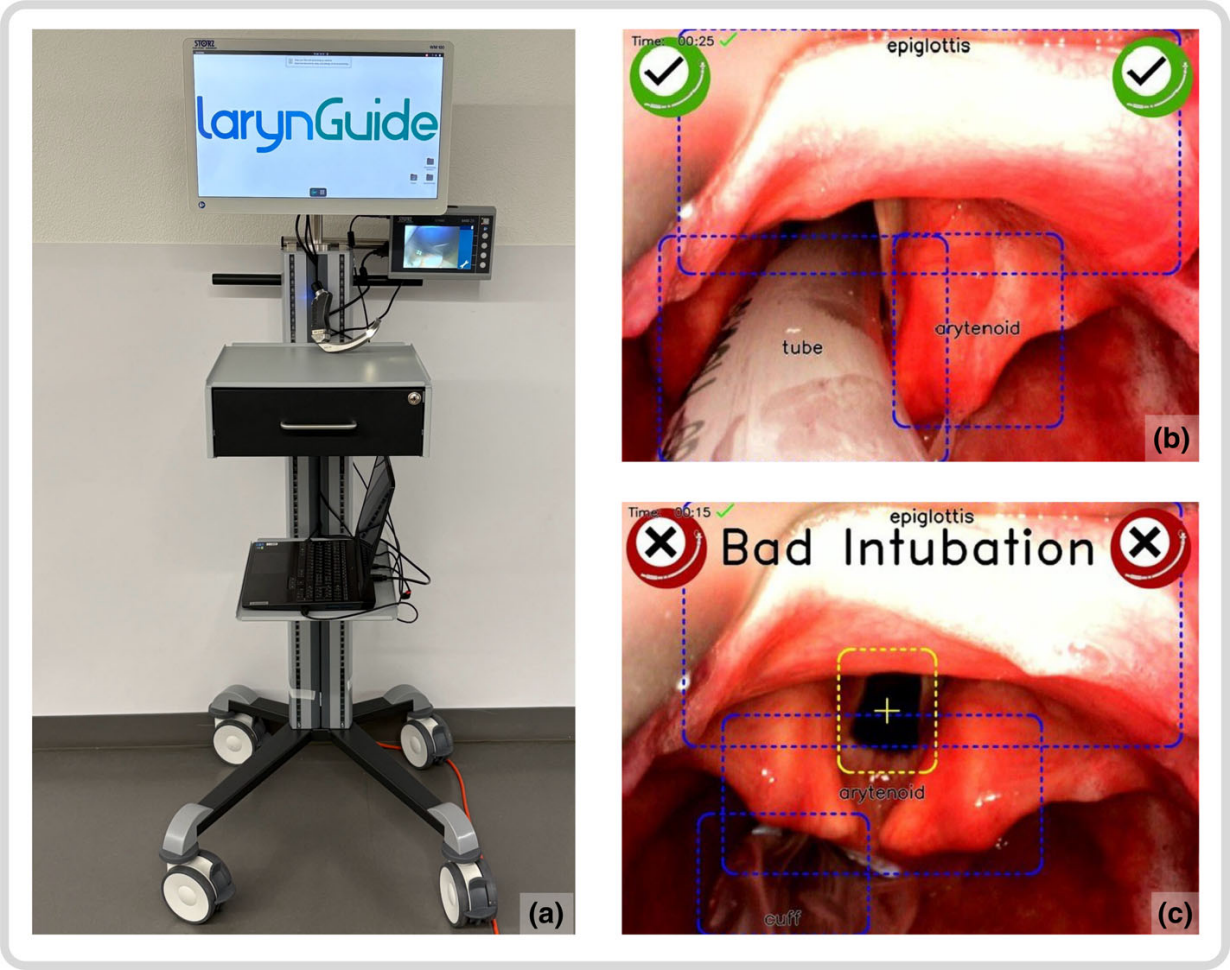
(a) Study setup with the screen of the videolaryngoscope and the larynGuide™ software running on a parallel screen. The software advice given on anatomical structures and the tracheal tube position was only visible on the study team's laptop, which was hidden from the airway operator. (b) Videolaryngoscopic view of the glottis. The software identified anatomical structures and tracheal tube position correctly with a green circle and a tick mark. (c) The software identified anatomical structures and the wrong tube position correctly, which was advised correctly with a red alert and a ‘bad intubation’ alert.

The larynGuide software was combined with a C‐MAC videolaryngoscope. The research setting consisted of two displays: a regular C‐MAC monitor, which the board‐certified anaesthetist used for tracheal intubation; and a second screen displaying the software for the study team to view that was not visible to the airway operator. Tracheal intubation was attempted in the supine position after standardised anaesthesia induction, including administration of a neuromuscular blocking drug with quantitative neuromuscular monitoring before tracheal intubation (train‐of‐four ratio of 0/4). After the tracheal intubation attempt, the airway operator assessed the tube position by a visual check of the tracheal tube through the vocal cords and measuring sustained exhaled carbon dioxide. In case of doubt, oesophageal intubation was ruled out after at least seven capnography waveforms [14]. The anaesthetist then informed the study team whether the placement of the tracheal tube was successful or not. Subsequently, the study team reviewed the advice from the larynGuide software about the tracheal tube position: a green circle with a tick mark for the correct position; or a red circle displaying the words ‘bad intubation’ (Fig. 1). The agreement between the airway operator and larynGuide confirmed accuracy.

Study termination criteria included unexpected difficult or impossible facemask ventilation; unplanned change to a hyperangulated blade; use of a bougie; or malfunction of the videolaryngoscope or the larynGuide software during the tracheal intubation attempt.

Primary outcome was accuracy of the advice on tracheal tube position given by the larynGuide software, measured by sensitivity (the proportion of correctly identified tracheal placements among those confirmed by the anaesthetist) and specificity (the proportion of correctly identified misplacements). Secondary outcomes included the first‐attempt tracheal intubation success rate; overall number of attempts; overall success rate of tracheal intubation; any technical problems of the larynGuide software; percentage of glottis opening (POGO) in five categories (0%, 1–25%, 26–50%, 51–75% and 76–100%); and incidence of complications during and after airway management.

We adopted the following hypothesis testing framework for the sensitivity (proportion of correctly indicated tube position) of the larynGuide software: H_0_: Se = Se_0_ and H_1_: Se > Se_0_. The reference sensitivity (Se_0_) was chosen to be 0.95, and we assumed a sensitivity of 0.995% of the larynGuide software (Se). A simulation was performed to determine the sample size, establishing that 110 patients would be required to show that the lower bound of the 95%CI of the estimated sensitivity is above the reference sensitivity with 90% power. The simulation was based on 10,000 random simulations with the assumptions above using Blaker's exact confidence interval for a binomial proportion. All statistical computations were performed in R 4.0.2 (R Foundation for Statistical Computing, Vienna, Austria).

We reported performance metrics of the larynGuide software (sensitivity; specificity; positive and negative predictive values; and diagnostic accuracy) as estimated mean (95%CI). Indeterminate outputs by the larynGuide software (e.g. no green circle or no ‘bad intubation’ alert) were considered test failures and were included as misclassifications in the analysis. Patients meeting termination criteria from the primary analysis were not included.

## Results

**Figure 2:**
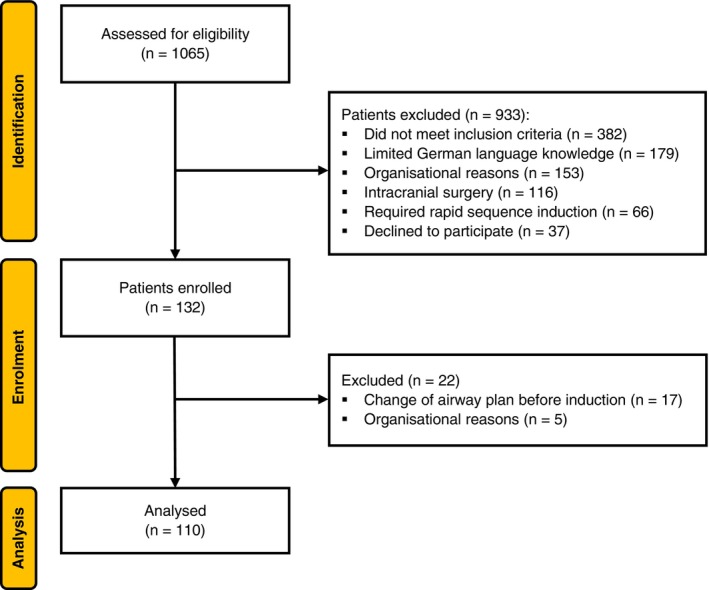
Patient recruitment flowchart.

From 31 October 2024 to 31 January 2025, we screened 1065 patients for eligibility and obtained written informed consent from 132, 110 of whom received the intervention and had complete datasets for analysis (Fig. 2). Patient characteristics are reported in Table 1.

**Table 1:** Patient characteristics. Values are number (proportion), mean (SD) or median (IQR [range]).

	Total
	n = 110
Sex; female	42 (38%)
Weight; kg	79 (18.2)
BMI; kg.m^‐2^	25.1 (22.9–29.1 [15.2–40.4])
Surgery	
Neurosurgery	12 (12%)
Ear, nose and throat	56 (51%)
Orthopaedic	42 (38%)
ASA physical status	
1	27 (25%)
2	58 (53%)
3	25 (23%)
History of previous difficult tracheal intubation	
Yes	1 (1%)
No	107 (97%)
Not available	2 (2%)
Induction drugs	
Propofol	110 (100%)
Ketamine	1 (1%)
Dexmedetomidine	8 (7%)
Opioids	
Fentanyl	102 (93%)
Sufentanil	6 (6%)
Remifentanil	56 (51%)
Neuromuscular blocking drug	
Rocuronium	106 (96%)
Succinylcholine	3 (3%)
Atracurium	1 (1%)

Tracheal tube placement was correct in 108 patients (98%) and incorrect in two (2%) as defined by the judgement of the board‐certified anaesthetist and by capnography (Table 2). Of the 108 correct placements, larynGuide detected 102 as correct, resulting in a sensitivity (95%CI) of 0.94 (0.88–0.98) (Table 3). The software misclassified tracheal tube position in six patients: two false negatives (the software advised a failed intubation despite correct placement); and four patients without any report provided due to fogging of the lens and poor image quality. The diagnostic accuracy (95%CI) was 0.94 (0.87–0.97). Among two patients of incorrect placement, one was correctly identified.

**Table 2:** Confusion matrix of the larynGuide™ compared with clinicians. Values are number (proportion).

	Successful tracheal intubation reported by larynGuide	Unsuccessful tracheal intubation reported by larynGuide	Total
Successful tracheal intubation	102 (93%)	6 (6%)^†^	108 (98%)
Unsuccessful tracheal intubation	1 (1%)	1 (1%)	2 (2%)
Total	103 (94%)	7 (6%)	110 (100%)

^†^ In two patients, larynGuide displayed the ‘bad intubation’ alert after the tracheal tube was positioned correctly in the trachea. In the other four patients, larynGuide did not report any outcomes.

**Table 3:** larynGuide™ performance metrics.

Performance metrics	Mean	95%CI
Sensitivity	0.94	0.88–0.98
Specificity	0.50	0.01–0.99
Diagnostic accuracy	0.94	0.87–0.97
Positive predictive value	0.99	0.95–1.00
Negative predictive value	0.14	0.00–0.58
Likelihood ratio: positive test	1.89	0.47–7.56
Likelihood ratio: negative test	0.11	0.02–0.54

First‐attempt tracheal intubation success rate was 106/110 (96%), and the tracheas of all patients were intubated successfully within three attempts (Table 4). Most patients (94, 84%) had a POGO score > 50% (Table 4). In the two patients where larynGuide displayed the ‘bad intubation’ alert incorrectly, the POGO scores were 51–75% and 76–100%, respectively. In the four patients where no advice was shown, the POGO scores were 76–100% (n = 2); 51–75% (n = 1); and 26–50% (n = 1).

**Table 4:** Tracheal intubation‐related data. Values are event rate (95%CI) or number (proportion).

	Total	Correct tracheal tube placement	Incorrect tracheal tube placement	p value
	n = 110	n = 108	n = 2	
**No. of attempts**				
1; n = 106	96% (91–99%)			
2; n = 3	3% (1–8%)			
3; n = 1	1% (0–5%)			
**Overall tracheal intubation success rate**	110 (100%)			
**Operator experience**				0.174
≥ 5 years	100 (91%)	99 (92%)	1 (50%)	
< 5 years	10 (9%)	9 (8%)	1 (50%)	
**POGO score**				0.001
76–100%	70 (64%)	70 (65%)	0	
51–75%	25 (23%)	25 (23%)	0	
26–50%	10 (9%)	10 (9%)	0	
1–25%	4 (4%)	3 (3%)	1 (50%)	
0	1 (1%)	0	1 (50%)	
**External laryngeal manipulation**				0.068
Not applied	81 (74%)	81 (75%)	0	
Applied	29 (26%)	27 (25%)	2 (100%)	

POGO, percent of glottic opening.

There were two adverse events during the study. One was a short period of oxygen desaturation where the patient's SpO_2_ dropped to 86% during the tracheal intubation attempt and recovered rapidly after tracheal intubation without any immediate or postoperative consequences. The other was an unplanned change of airway management strategy after an unsuccessful first tracheal intubation attempt. The second attempt was successful using a bougie (Frova Intubating Introducer, Cook Medical, Limerick, Ireland).

After viewing the results and in consultation with the manufacturer of the software, it was noticed that in 14 patients, non‐standard tracheal tubes were used (i.e. coil spring tube; tubes with a neuromuscular transmission electrode; and microlaryngeal tubes), for which the larynGuide software was not trained for specifically. Not including these patients in the analyses resulted in a sensitivity (95%CI) of 0.96 (0.89–0.99) and in diagnostic accuracy (95%CI) of 0.95 (0.88–0.98). Specificity (95%CI) remained unchanged at 0.50 (0.01–0.99).

## Discussion

This prospective observational study is the first to investigate the accuracy of larynGuide software for the correct tracheal tube position in humans. The software had a diagnostic accuracy of 94% with a sensitivity of 94% in identifying correct tracheal tube placement, which was below the targeted 95% sensitivity. The specificity of the software remained at 50%, indicating an important limitation in recognising oesophageal intubation and limiting its current application in clinical practice. These findings align with recent advancements in AI‐driven airway management, where machine learning models have improved tracheal intubation success rates and predictive accuracy [15]. However, it is important to recognise that larynGuide cannot replace established confirmation techniques like capnography, which remain the gold standard due to their higher sensitivity and reliability. Rather, its clinical value may lie in the guidance of visual confirmation, especially in routine tracheal intubation with optimal visualisation.

Tracheal intubation is crucial in various medical disciplines, including anaesthesia; critical care; and emergency medicine. Videolaryngoscopy improves visualisation of the glottis and reduces oesophageal intubation rates [6, 7]. However, challenges persist, particularly among less experienced airway providers, in patients presenting with a difficult airway. Identifying the correct tracheal tube position can be challenging for airway providers. The integration of an AI‐based decision support tool, such as larynGuide, could refine airway management further. In this study setting, the software was particularly effective when visibility of the glottis was good, highlighting the potential benefit of AI‐assisted laryngoscopy in routine settings. However, in the six patients where the software failed (i.e. no green circle or ‘bad intubation’ alert), impaired visualisation due to fogging of the camera lens or poor image quality was identified as a limiting factor. Unfortunately, from a clinician's perspective, these are the scenarios where decision‐making is particularly challenging and an additional tool might be helpful in a time‐critical situation.

Previous research indicated that technological improvements, such as anti‐fogging mechanisms and higher‐resolution imaging, may enhance AI‐based software performance in airway management further [16], and have shown the potential of AI in improving laryngeal visualisation and tracheal intubation success. Masumori et al. developed glottic recognition software using a combination of deep learning models, achieving over 95% accuracy in localising the vocal cords and epiglottis during videolaryngoscopy [16]. However, that study was conducted in manikins and a controlled environment. In contrast, our study evaluated larynGuide during tracheal intubations in humans having elective surgery, incorporating all potential real‐world challenges such as secretions; anatomical variations; limited visibility; and the different approaches and experience of videolaryngoscopy of airway providers. The fact that larynGuide achieved an overall sensitivity of 94%, only slightly lower than the AI‐based software model of Masumori et al. despite these additional complexities, underscores the efficiency and robustness of larynGuide for potential clinical use. Similarly, Khan et al. reviewed the role of AI in robotic‐assisted tracheal intubation, emphasising its potential to reduce operator dependency and improve procedural precision [17]. The performance of larynGuide aligns with these studies, particularly its ability to assist clinicians by providing real‐time guidance. However, compared with the automated robotic systems explored by Biro et al., which showed successful automated tracheal intubation in manikins with a 98–100% success rate, our system still requires further optimisation for real‐world clinical application [13].

A comparison with the study by Matava et al. highlights key differences in AI applications for airway management [18]. In that study, convolutional neural networks were employed to classify and label vocal cords and tracheal anatomy during laryngoscopy and bronchoscopy in patients with three different models, achieving a sensitivity of 85%, 76% and 56%, depending on the model used. The corresponding specificity was 98%, 98% and 85%, respectively. While their AI model showed effective classification, it focused primarily on video‐based airway recognition rather than real‐time tracheal intubation guidance. In contrast, larynGuide integrates AI‐driven feedback to guide tracheal tube placement in real‐time, bridging the gap between recognition and action. Notably, larynGuide achieved an overall sensitivity of 94%, highlighting its strong performance compared with other published models, despite real‐world challenges. However, the lower specificity of larynGuide suggests that additional refinement is needed to match the higher accuracy reported in manikin studies, potentially by incorporating deep learning techniques [16, 18]. One possible explanation for the low specificity is that in the two patients where the tracheal tube slipped paratracheally, the tracheal tube was retracted abruptly by the operator, potentially not giving the software enough time for diagnostics.

Our study has several limitations. It was observational and conducted in a single centre with only one device and standard blades evaluated, which may limit the generalisability of our findings to other clinical environments and patient populations. The high first‐attempt tracheal intubation success rate in our cohort (96%) limited the number of incorrect intubations, thereby constraining the ability to evaluate robustly the ‘bad intubation’ alert function of larynGuide under real‐world failure conditions. Additionally, we focused only on the ability of the software to recognise tracheal tube position rather than its role in guiding tracheal tube placement. This decision was made because it was the first time the software was used on humans, and we prioritised evaluating its accuracy in tracheal tube recognition before assessing its real‐time guidance capabilities. As a result, the study does not provide insights into the effectiveness of the software in assisting with the tracheal intubation procedure itself. Future randomised controlled trials will be necessary to evaluate its potential in guiding tracheal intubation. Notably, larynGuide is not intended to replace established confirmation methods such as capnography but may serve as a supportive tool in airway management. As this study included primarily patients having elective surgery with expected normal airways, generalisability to emergency or high‐risk populations is limited. Despite its promising sensitivity, larynGuide exhibited low specificity, highlighting limitations in detecting incorrect tube placements. This may be related to image quality issues such as fogging, suboptimal views or use of non‐standard tracheal tubes [17]. These findings underscore the need to expand the training data in the system to accommodate a wider range of airway conditions and equipment [19]. As the software was not updated during the study, its potential for continuous learning and adaptation was not assessed. Future research should evaluate whether iterative updates and technical refinements can enhance diagnostic performance under varied clinical conditions.

This observational study reports the first in‐human use of larynGuide, an AI‐driven software for advice on tracheal tube position. It established a sensitivity of 94% in identifying tracheal tube placement correctly. While this is promising for its potential as an adjunctive tool in airway management, it does not match the diagnostic reliability of standard confirmation methods such as capnography and cannot be used as a standalone solution. Despite these challenges, the software performed promisingly in real‐life clinical scenarios, reinforcing the feasibility of AI‐assisted tracheal intubation.
